# The Association of Serum Erythroferrone, a Regulator of Erythropoiesis and Iron Homeostasis, with Cardiometabolic Risk Factors in Apparently Healthy Young Adults—A Preliminary Study

**DOI:** 10.3390/nu17203205

**Published:** 2025-10-12

**Authors:** Katarzyna Bergmann, Anna Stefańska, Magdalena Krintus

**Affiliations:** Department of Laboratory Medicine, Collegium Medicum in Bydgoszcz, Nicolaus Copernicus University in Toruń, 87-100 Toruń, Poland; zuzanna@cm.umk.pl (A.S.); krintus@cm.umk.pl (M.K.)

**Keywords:** erythroferrone, iron homeostasis, cardiometabolic risk, overweight

## Abstract

**Background**: Recent studies suggest that erythroferrone (ERFE), an iron-regulating protein whose primary role is to inhibit hepcidin synthesis, may affect glucose and lipid metabolism, and its serum concentration is reduced in obese and diabetic individuals. The aim of this study was to evaluate the association of ERFE concentration with selected cardiometabolic risk factors in apparently healthy young adults. **Methods**: This preliminary study consisted of 122 (63 females, 59 males) normoglycemic, non-smoking subjects aged 25–40 years. In all participants, anthropometric measurements and the following laboratory tests were performed: fasting plasma glucose, glycated hemoglobin (HbA1c) and serum iron, lipid profile, insulin, Homeostatic Model Assessment for Insulin Resistance (HOMA-IR), C-reactive protein (CRP), ERFE and hepcidin. **Results**: The serum ERFE concentration was significantly lower in men compared to women (*p* = 0.009) and in subjects who were overweight (*p* < 0.001) and had abdominal obesity (*p* < 0.001). ERFE showed significant negative correlations with body mass index, waist–hip ratio, HbA1c, CRP, insulin, HOMA-IR and triglycerides. In the logistic regression analysis, ERFE was significantly associated with being overweight (OR = 0.051; *p* = 0.004), abdominal obesity (OR = 0.372; *p* < 0.001), HOMA-IR ≥ 2.0 (OR = 0.584; *p* = 0.013), CRP > 1 mg/L (OR = 0.648; *p* = 0.020) and triglycerides (OR = 0.521; *p* = 0.033). A relevant predominance in the prevalence of cardiometabolic risk factors was observed in subjects with ERFE levels in the first tertile (<1.35 ng/mL), compared to the third tertile (>2.19 ng/mL). **Conclusions**: Serum ERFE is inversely associated with being overweight, increased waist circumference, CRP, and markers of insulin resistance and lipid abnormalities, suggesting its potential relevance as a marker of early cardiometabolic risk in apparently healthy young adults.

## 1. Introduction

The role of iron in the pathophysiology of cardiometabolic disorders has been the subject of intensive research in recent decades. In individuals with obesity and metabolic syndrome (MetS), elevated levels of ferritin and hepcidin—key proteins involved in iron metabolism and storage—are commonly observed [1]. This association is explained by the proinflammatory activity of adipose tissue, which, by producing proinflammatory cytokines, particularly interleukin-6 (IL-6), strongly stimulates hepcidin synthesis in the liver. This condition, on the one hand, reduces iron availability in plasma leading to impaired erythropoiesis and anemia, and on the other hand, it promotes excessive iron accumulation in hepatocytes and macrophages, inducing oxidative stress and inflammation and thereby contributing to metabolic disorders such as insulin resistance and dyslipidemia [2].

Erythroferrone (ERFE) is an iron-regulating protein produced by erythroblasts in response to erythropoietin (EPO). Its primary role is to inhibit hepcidin synthesis, which increases iron availability for erythropoiesis. ERFE was initially identified in 2012 as myonectin (also known as C1q/TNF-related protein 15, CTRP15) in a mouse model study [3]. It was described as a protein secreted by skeletal muscle, particularly in response to physical activity and food intake promoting fatty acid uptake by the liver and adipose tissue and exerting potentially beneficial metabolic effects, such as reducing insulin resistance and fatty liver development. As a regulator of erythropoiesis, ERFE was first demonstrated in 2014 in a study by Kautz et al. [4]. In a mouse model, increased mRNA expression of the Fam132b gene was observed in bone marrow erythroblasts and in the spleen in response to phlebotomy and EPO stimulation, leading to a decrease in hepcidin synthesis in hepatocytes. ERFE has been shown to be an important link regulating hepcidin levels in response to increased iron demand during erythropoiesis caused by, among others hemorrhage, intensive growth or anemia. By reducing hepcidin production and thus iron accumulation in tissues, ERFE may potentially reduce oxidative and inflammatory reactions at the cellular level and improve metabolic pathways related to the utilization of glucose and fatty acids [5].

Despite differences in functional context, myonectin and erythroferrone are considered to be the same protein, encoded by the same gene and sharing a characteristic structure of the CTRP family [6]. ERFE expression in non-erythropoietic tissues, such as the liver skeletal muscle and adipose tissue, is relatively low compared to the bone marrow and spleen, suggesting that ERFE may also participate in pathways beyond iron regulation [5]. However, there are a limited number of studies examining the relationship between ERFE levels and metabolic disorders. There is also a particular lack of data from healthy individuals that, from a broader perspective, could be helpful in evaluating the diagnostic value of ERFE as an early cardiometabolic risk factor. The aim of this preliminary study was to assess the relationship between serum ERFE concentration, anthropometric measurements and selected laboratory indicators of glucose and lipid metabolism in apparently healthy, normoglycemic young adults.

## 2. Materials and Methods

### 2.1. Study Group

This preliminary study included 122 apparently healthy, white European adults (63 females, 51.6%) aged 25–40 years, selected from the general population. All subjects completed a standard medical questionnaire regarding their health status. The following exclusion criteria from the study were applied: smoking, obesity (body mass index, BMI ≥ 30 kg/m^2^), impaired fasting glucose (≥5.55 mmol/L; ≥100 mg/dL) or diabetes, hypertension (≥140/≥90 mmHg), decreased (<11.6 μmol/L for males and <8.9 μmol/L for females) or elevated (>31.3 μmol/L for males and >30.3 μmol/L for females) serum iron concentration [7], thyroid disease, renal disease, liver disease, pregnancy, acute infection in the 3 weeks prior to the study, chronic inflammatory and autoimmune diseases, history of cardiovascular disease and cancer. For a more reliable translation of the results to the general population, the study included 30 (24.6%) overweight subjects, which is a common phenomenon in the Polish population and affects approximately 38% of adults [8]. However, individuals with clinically defined obesity were excluded, as it clearly disturbs metabolic processes and is usually associated with diagnosed comorbidities. All participants declared good health and no use of any medications, including hormonal birth control in the case of women.

The study was conducted in accordance with the Declaration of Helsinki, and approved by the Bioethics Committee of Nicolaus Copernicus University in Torun, Collegium Medicum in Bydgoszcz, Poland (no. KB/627/2010, annexed 23 April 2013). Written consent was obtained from all participants before inclusion in the study.

### 2.2. Laboratory and Anthropometric Measurements

Prior to blood sampling, basic anthropometric measurements were performed in all participants: body weight, waist circumference (WC) and hip circumference, based upon which BMI and waist–hip ratio (WHR) were calculated. Being overweight was defined as having a BMI value in the range of 25.0–29.9 kg/m^2^. An increased waist circumference, indicating abdominal obesity, was defined as being ≥80 cm in women and ≥94 cm in men, respectively [9]. Blood pressure measurements were taken in a sitting position, after at least 15 min of rest, in three repetitions at 2–3 min intervals, using an automatic M6 Comfort blood pressure monitor (Omron Healthcare, Kyoto, Japan). Acceptable mean blood pressure values were <140 mmHg for systolic blood pressure (SBP) and <90 mmHg for diastolic blood pressure (DBP). An elevated blood pressure was defined as a SBP of 120–139 mmHg or DBP of 70–89 mmHg [10].

Fasting venous blood samples were collected in the morning (7.00–9.00 a.m.), after at least 10 h of fasting, using a disposable Vacutainer system (Becton Dickinson, Franklin Lakes, NJ, USA) into a 6 mL tube with clot activator to obtain serum for biochemical measurements, two 2 mL tubes containing EDTA for glycated hemoglobin (HbA1c) and sodium fluoride for plasma glucose measurements. Samples with clot activator were left for 30 min at room temperature to clot completely and then centrifuged at 3000 rpm for 15 min. Samples for glucose testing were centrifuged immediately after collecting at 3000 rpm for 15 min at 4 °C.

Concentrations of plasma glucose, HbA1c in the whole blood, serum lipid profile, C-reactive protein (CRP), iron (Fe) and insulin were measured in fresh samples. The remaining serum was transferred in small aliquots to cryotubes and frozen at −80 °C until ERFE and hepcidin assays were performed. All basic biochemical measurements were performed on the ARCHITECT ci8200 platform (Abbott Laboratories, Chicago, IL, USA). The concentration of serum ERFE was measured using a commercially available kit (ref. ELK7647, ELK Biotechnology CO., Ltd.; Wuhan, China), based on the sandwich enzyme-linked immunosorbent assay (ELISA) with a detectable range of 0.16–10.0 ng/mL. Hepcidin concentration in the serum was determined using a highly sensitive competitive ELISA method (ref. EIA-5782, DRG International, Inc., Springfield, NJ, USA) with a detectable range of 0.153–81.0 ng/mL. Before testing, samples were thawed gradually at −21 °C, 4 °C and room temperature, mixed thoroughly and centrifuged at 5000 rpm for 5 min.

Low-density lipoprotein cholesterol (LDL-C) was calculated using the Sampson formula [11], while non-high-density lipoprotein cholesterol (non-HDL-C) was determined by subtracting high-density lipoprotein cholesterol (HDL-C) from total cholesterol (TC). Homeostatic Model Assessment for Insulin Resistance (HOMA-IR) values were calculated using following equation: HOMA-IR = [glucose (mmol/L) ×insulin (µU/mL)]/22.5.

### 2.3. Statistical Analysis

Statistical analysis was performed using Statistica 13.3 software (StatSoft Inc., Tulsa, OK, USA). Normality was assessed using the Shapiro–Wilk test and data were presented as the median with Q1–Q3 ranges (25th–75th percentile) for non-Gaussian distributions. Comparisons between two groups were performed using the Mann–Whitney U test. Spearman rank correlation coefficients were calculated between ERFE and other variables. The chi-square (Fisher exact) test was used to evaluate differences between two percentage values or correlation coefficients. Logistic regression was used to assess the association between ERFE concentrations and cardiometabolic risk factors, with odds ratios calculated per unit (1 ng/mL) increase in ERFE concentration. A *p*-value of <0.05 was considered to be statistically significant.

## 3. Results

### 3.1. General Characteristics of the Study Group

Clinical and biochemical characteristics of the study group are presented in Table 1. Compared to men, women had significantly lower values of BMI, waist circumference and WHR, blood pressure, iron, hepcidin, fasting plasma glucose, insulin, HOMA-IR, triglycerides (TG), non-HDL-cholesterol (non-HDL-C) and apolipoprotein B (apoB), whereas HDL-C and apolipoprotein AI (apoAI) were higher. Serum ERFE concentration was also significantly higher in women than in men (*p* = 0.009). The prevalence of being overweight and abdominal obesity was higher in men, although these differences were not statistically significant.

**Figure 1:**
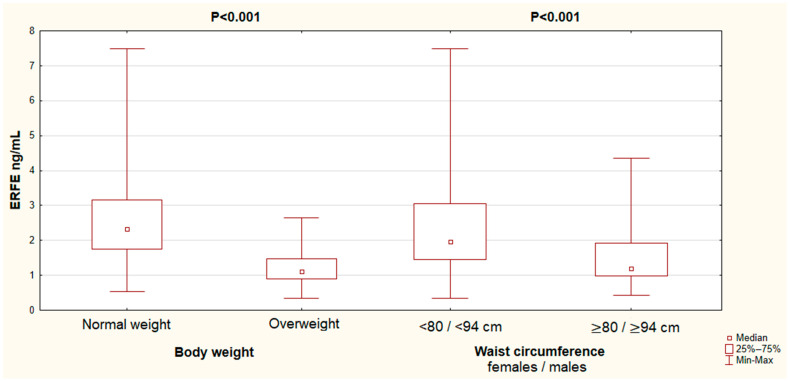
ERFE concentrations in serum according to body weight status and waist circumference.

Additionally, ERFE concentrations were analyzed according to body weight and waist circumference (Figure 1). Significantly lower ERFE levels were found in both overweight individuals (median 1.11 vs. 2.33 ng/mL; *p* < 0.001) and those with an increased waist circumference (≥80 cm for women and ≥94 cm for men), indicating abdominal obesity (median 1.19 vs. 1.97 ng/mL; *p* < 0.001), compared to individuals with a normal weight and waist circumference. When analyzing iron-related indicators, hepcidin concentrations were significantly higher in subjects who were both overweight (median 14.3 vs. 28.0 ng/mL; *p* < 0.001) and had abdominal obesity (median 18.0 vs. 23.6 ng/mL; *p* = 0.036) compared to lean participants, while serum iron levels did not differ significantly (data not presented).

### 3.2. Correlation Analysis

Correlation analysis (Table 2) indicated that ERFE concentration in the study group showed significant negative correlations with age; anthropometric indices, particularly BMI (R = −0.65; *p* < 0.001); CRP; HbA1c; insulin; HOMA-IR and triglycerides, and weak but statistically relevant correlations with systolic blood pressure, non-HDL-C and apoB. A positive correlation was observed only for HDL-C. After dividing the group by sex, the correlation coefficients became weaker and did not differ significantly between women and men. However, in women, stronger correlations compared to men were observed for waist circumference, WHR and CRP. Among the lipid parameters, in women, ERFE correlated only with TG. In men, strongest correlations were noted for BMI, waist circumference and systolic blood pressure, and also for TG, non-HDL-C and apoB. Considering the indicators of iron homeostasis, ERFE showed a significant negative correlation with hepcidin in both women and men, while a positive correlation with serum iron concentration was observed only in men (Figure S1, Supplementary Material). Contrary to ERFE, hepcidin correlated positively with TG (R = 0.35; *p* < 0.001), non-HDL-C (R = 0.25; *p* = 0.006), CRP (R = 0.29; *p* = 0.006) and waist circumference (R = 0.52; *p* < 0.001). However, compared to ERFE, hepcidin displayed a weaker correlation with BMI (R = 0.45; *p* < 0.001) and no significant relationship with HbA1c and HOMA-IR, but stronger correlations with WHR (R = 0.51; *p* < 0.001), HDL-C (R= −0.41; *p* < 0.001) and apoB (R = 0.30; *p* = 0.001).

### 3.3. Association of ERFE Concentration with Cardiometabolic Risk Factors

In a further analysis, ERFE concentration was assessed as a predictor of cardiometabolic risk factors (Table 3). ERFE was inversely associated with being overweight and abdominal obesity, but also with the occurrence of hypertriglyceridemia of ≥1.69 mmol/L (≥150 mg/dL), early insulin resistance defined by HOMA-IR > 2.0 and a CRP level > 1 mg/L, considered to be a moderate/high cardiovascular disease (CVD) risk factor. However, no significant relationship was found between ERFE and the occurrence of elevated blood pressure or decreased HDL-C concentration. In models adjusted for age and sex, ERFE remained a significant predictor for being overweight, abdominal obesity and having a CRP level of >1 mg/L, but not for hypertriglyceridemia. After additional adjustment for BMI, no significant associations were found.

**Figure 2:**
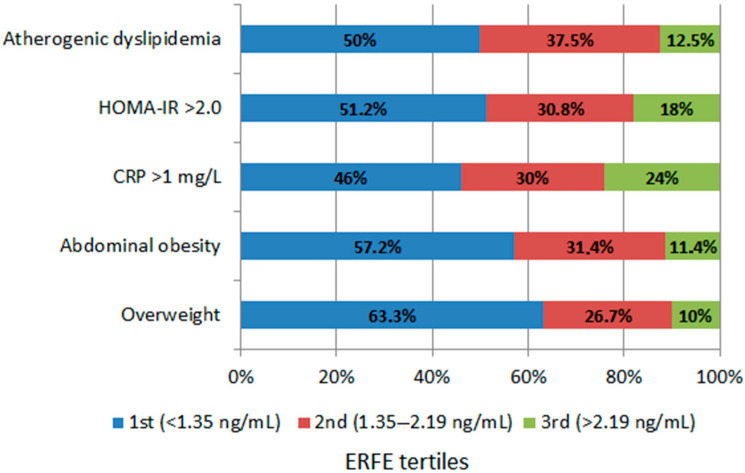
Prevalence of selected cardiometabolic risk factors across tertiles of ERFE concentration.

The incidence of selected cardiometabolic disorders varied depending on the tertile of ERFE concentration (Figure 2). In the first tertile (<1.35 ng/mL), a significant predominance was observed in the percentage of individuals who were overweight (63.3% vs. 10%; *p* < 0.001), had abdominal obesity (57.2% vs. 11.4%; *p* < 0.001) and had a HOMA-IR > 2.0 (51.2% vs. 18%; *p* = 0.021) compared to subjects in the third tertile. Among participants with a moderate/high CVD risk, defined as CRP > 1 mg/L, the percentage of subjects in the first tertile of ERFE was almost twice as high as in the third tertile (*p* = 0.021). Although atherogenic dyslipidemia, defined according to the Adult Treatment Panel III (ATPIII) as the coexistence of elevated TG > 1.69 mmol/L (>150 mg/dL) and decreased HDL-C < 1.03 mmol/L (<40 mg/dL) [12], was rare in the study group (n = 8, 6.6%), half of the cases concerned subjects with an ERFE concentration in the first tertile.

## 4. Discussion

Our study demonstrated that serum erythroferrone concentration is inversely correlated with BMI, waist circumference, CRP, HOMA-IR and lipid profile components. The strong negative correlation between ERFE and BMI suggests that changes in body composition, particularly body fat content, may influence serum ERFE concentrations. However, an open question remains in order to find a causal relationship between serum ERFE and being overweight/obesity and to clarify whether its reduced concentration leads to metabolic disorders or is a secondary effect of insulin resistance, inflammation and other mechanisms related to excessive adipose tissue. It seems equally important to investigate whether an increase in ERFE concentration may translate into an improvement in the metabolic profile or demonstrate a direct protective effect and reduction in cardiometabolic risk. Our preliminary results showed that ERFE is a significantly associated with being overweight and selected cardiometabolic risk factors in the univariate logistic regression analysis; however, multivariable models adjusted for sex, age and BMI did not confirm that ERFE can be considered to be an independent predictor. Available publications from the scientific literature on ERFE focus primarily on its role in the regulation of iron concentration and the process of erythropoiesis. However, emerging evidence, including findings from animal studies, suggests a potential relationship between ERFE and alterations in body composition, as well as glucose and lipid metabolism. Although the exact mechanisms remain unclear, many results indicate a potential protective role of ERFE in metabolic processes, shedding new light on the link between iron homeostasis and cardiometabolic risk.

In a study by Seldin et al., ERFE (described as myonectin) was identified as a myokine, whose mRNA expression and serum concentration were closely related to alterations in the energy state in mice myocytes [3]. Serum myonectin decreased in diet-induced obesity, as well as during fasting, but increased in response to physical exercise and feeding. Administration of recombinant myonectin reduced the free fatty acid concentration in the blood without altering adipose tissue lipolysis. Moreover, it was suggested that the postprandial rise in myonectin concentration after a meal may be caused by both the stimulation of glucose and lipid uptake in muscle tissue, as well as incretin hormones such as glucagon-like protein-1 (GLP-1), which additionally support energy metabolism. Although the authors of the study focused exclusively on the role of myonectin/ERFE produced in muscles and did not address the importance of myonectin/ERFE in the regulation of iron homeostasis, this does not exclude the possibility of a similar effect on glucose and lipid metabolism by ERFE produced by erythroblasts, which constitute the largest part of its concentration in plasma.

A limited number of human studies indicate that ERFE levels may be lower in individuals with cardiometabolic disorders. A study of 362 Chinese individuals with type 2 diabetes showed that the serum ERFE/myonectin concentration was significantly lower compared to that of healthy controls [13]. In the non-diabetic group, obese individuals also had reduced ERFE levels compared to lean participants. ERFE concentrations were negatively correlated with BMI, TG, LDL-C, CRP, HbA1c, insulin, HOMA-IR and both the visceral and subcutaneous fat area in diabetic patients. Hilton et al. [14] reported that plasma ERFE is weakly negatively correlated with BMI and android-to-total-fat ratio in apparently healthy women, but not in men. ERFE was also negatively correlated with TG in both sexes after adjustment for BMI. Another study found serum myonectin/ERFE concentrations to be lower in obese patients compared to controls, rising six months after sleeve gastrectomy [15]. Similarly, ERFE correlated negatively with anthropometric indices, glucose, HOMA-IR and HbA1c. On the contrary, higher levels of plasma myonectin/ERFE were found in individuals with type 2 diabetes and prediabetes in a study by Li et al. [16]; furthermore, positive correlations with WHR, body fat and indicators of glucose metabolism were observed, suggesting that myonectin/ERFE is a predictor of diabetes development. Nevertheless, the authors did not clearly explain the association between the increased risk of diabetes and impaired glucose tolerance, and plasma ERFE concentration. One of the hypotheses presented was that increase myonectin/ERFE in subjects with diabetes and impaired glucose tolerance might be an effect of a response to metabolic stress from resistance to myonectin action or dysregulation of myonectin signaling and synthesis in response to hyperinsulinemia and hyperglycemia related to insulin resistance. However, the authors did not demonstrate a significant increase in myonectin/ERFE concentration in response to increased glucose and insulin concentrations in both the oral glucose tolerance test and the euglycemic–hyperinsulinemic clamp test. The differences in the results of this study may be related to study limitations, including a relatively small and ethnically homogeneous study group and methodological factors, i.e., the types of reagents used for the ERFE assay.

Interestingly, our study revealed significant differences in serum ERFE concentrations between women and men. Compared to men, women had a better metabolic profile, including significantly lower values of anthropometric indices, blood pressure, glucose, HOMA-IR and proatherogenic components of the lipid profile. The literature still lacks an in-depth analysis explaining sex differences in ERFE concentration, and the results of some studies are inconsistent. A study by Appleby et al. [17] in 155 healthy subjects showed significantly higher ERFE concentration in men compared to women (0.67 vs. 0.32 ng/mL; *p* < 0.001), while Ganz et al. [18] did not indicate relevant differences between males and females (12 vs. 11 ng/mL). In The CHRIS study [19], the serum ERFE concentration changed in different age categories, reaching similar values in women and men aged 30–39, with significantly higher values in women aged 40–49 and decreasing after the age of 50. At this point, it is worth considering potential factors determining the higher ERFE concentration in women from our cohort. On the one hand, this phenomenon may be related to lower adiposity in women, especially visceral, than in men, which is indirectly indicated by a lower BMI and WHR values in women. As mentioned above, few human studies have shown a significant inverse association between ERFE and BMI and obesity [13,14,15]. Although our study group included non-obese individuals with a BMI not exceeding 28.3 kg/m^2^, an increased waist circumference, observed more frequently in men (35.6%), and the accumulation of visceral fat tissue are factors strongly determining unfavorable metabolic changes that may potentially also be reflected in iron homeostasis parameters, including ERFE. On the other hand, it is worth paying attention to the possibility of increased stimulation of ERFE secretion in women, related to iron loss during the menstrual cycle, independently of metabolic status. Additionally, in our cohort, we observed a positive correlation between ERFE and iron in men, but not in women. Median iron and hepcidin concentrations were significantly lower in women when compared to men. These findings may also indicate a significant influence of the menstrual cycle as a confounding factor—in men, due to greater iron reserves and availability, erythropoietic stimulation is weaker than in women, which may result in lower serum ERFE concentrations. To our knowledge, there have been no studies to date on changes in ERFE levels during the menstrual cycle. However, we hypothesize that, like with iron, ferritin or hepcidin concentrations, it may fluctuate depending on the phase of the menstrual cycle. During menstrual bleeding in the follicular phase, the concentrations of iron, ferritin and hepcidin are lower and increase during the ovulation phase [20]. Therefore, ERFE levels may be higher in the first half of the cycle in response to iron loss, and decrease during ovulation and the luteal phase. In our study, we did not record the day of the cycle on which women reported for blood sampling, which potentially complicates the assessment of the relationship between ERFE, iron parameters and cardiometabolic risk factors. Female participants were informed not to collect blood samples during menstruation and within 3–5 days afterwards to prevent possible interference with laboratory test results, which could partially mitigate the discrepancies in ERFE determinations. Nevertheless, this issue requires further investigation to assess the influence of hormonal changes on the reliability of serum ERFE concentrations.

The direct mechanism linking ERFE with cardiometabolic risk remains unknown; however, several hypotheses need to be investigated. Iron is a nutrient essential for energy metabolism, including ATP synthesis, yet disturbances in iron homeostasis and metabolic disorders are closely interlinked. As mentioned in the introduction, ERFE is a main regulator of hepcidin synthesis and iron availability in plasma; therefore, a decreased ERFE concentration may lead to excessive iron storage in tissues, including the liver, muscle and adipose tissue. Due to the high oxidative potential of iron, this may result in oxidative stress, lipoxygenation and mitochondrial dysfunction, which promote insulin resistance, dyslipidemia and other disorders associated with metabolic syndrome [21]. Indirect effects may be mediated via adipocytes—iron-induced inflammation and oxidative stress can modulate the secretion of proinflammatory adipocytokines, which in turn affects insulin sensitivity and lipoprotein production [1,21]. Hepcidin, the key iron-regulatory hormone, has been consistently associated with obesity, visceral fat, dyslipidemia, inflammation, type 2 diabetes and cardiovascular disease [22,23,24]. Its levels rise with the number of metabolic syndrome components [25], largely due to adipocyte-driven low-grade inflammation and IL-6–mediated stimulation of hepatic hepcidin synthesis [26]. Hepcidin can also be produced in adipose tissue, further disrupting iron and energy balance [27]. Dysmetabolic iron overload syndrome (DIOS), characterized by moderate hepatic iron excess, elevated ferritin and hepcidin, and metabolic dysfunction, exemplifies this link [28,29]. In this context, ERFE, as a potent hepcidin suppressor, may influence adipose tissue activity, inflammation and oxidative stress. Our study strengthens this hypothesis by showing a negative correlation between ERFE and hepcidin, while hepcidin correlated positively with body weight, CRP and lipid indices. However, these assumptions do not clearly explain whether higher ERFE concentrations can act directly at the tissue and receptor levels to improve energy metabolism. A more likely hypothesis seems to be that ERFE may be part of a more complex mechanism related to EPO activity, which has demonstrated beneficial metabolic effects in numerous studies in both experimental models and humans, including reduced insulin resistance and improved lipid and adipocytokine profiles [30,31,32]. However, contrary observations from the study on ERFE-knockout mice (*Erfe^-/-^*), where the lack of ERFE secretion did not significantly alter insulin sensitivity under standard conditions and after EPO stimulation, suggest that ERFE is not necessary for the action of EPO in regulating glucose metabolism [33]. Therefore, it is crucial to extend the research to determine the exact molecular pathways of ERFE in the pathomechanism of metabolic dysfunctions.

Recent findings support the broader relevance of ERFE beyond erythropoiesis. In healthy men, physical training was shown to increase circulating ERFE together with EPO, indicating its responsiveness to lifestyle and physiological stressors [34]. Conversely, in high-risk populations such as patients with chronic kidney disease, elevated ERFE has been associated with mortality and cardiovascular events, especially under therapy with erythropoiesis-stimulating agents [35]. Experimental studies in humans demonstrated that ERFE rises rapidly after recombinant EPO administration or high-altitude exposure, accompanied by a decline in hepcidin, highlighting its role as a sensitive biomarker of erythropoietic activity. At the mechanistic level, in vitro data suggest that ERFE may modulate bone morphogenetic protein-6 (BMP6) signaling in hepatocytes, influencing not only hepcidin, but also genes involved in lipid metabolism, such as fatty acid transporters [36]. However, animal models point to context-dependent effects: overexpression of ERFE causes iron overload and metabolic abnormalities [37], while ERFE-knockout mice retain EPO-induced improvements in insulin sensitivity [33].

Taken together, these studies support a complex, potentially dual role of ERFE in iron and metabolic regulation, consistent with our finding that lower ERFE levels were associated with being overweight, abdominal obesity and insulin resistance in apparently healthy young adults. To our knowledge, this is one of the first published studies assessing the relationship between ERFE and cardiometabolic risk factors in apparently healthy individuals, highlighting the potential relevance of serum ERFE as a marker of early cardiometabolic risk. However, several important limitations of study require comment. The sample size is very small and represents a homogeneous group in terms of ethnicity and age; therefore, our findings may not be directly applicable to all populations. We included only individuals with a normal body weight or who were overweight, which does not allow us to show possible relationship or differences in the association of ERFE with metabolic disorders in subjects already diagnosed with obesity. We also did not provide data on menstrual cycle phase in female participants, which may be a confounding factor for serum ERFE concentrations. Finally, the cross-sectional design limits our ability to infer a causal relationship between serum ERFE and cardiometabolic risk. 

## 5. Conclusions

Our study demonstrated a significant association between serum ERFE concentration and cardiometabolic risk factors, particularly overweight, markers of insulin resistance and inflammation, and lipid abnormalities, in young, apparently healthy, normoglycemic adults. This indicates the potential importance of ERFE measurements as an indicator of early metabolic disorders. Despite the promising findings, our results should be considered preliminary and warrant confirmation in larger, population-based cohorts.

## Figures and Tables

**Table 1:** Clinical and biochemical characteristics of study group.

Variables	All (*n* = 122)	Women (*n* = 63)	Men (*n* = 59)	*p*
Age (years)	30.5 (26–34)	31 (25–36)	30 (27–34)	0.587
BMI (kg/m^2^)	23.8 (21.1–26.1)	21.4 (20.1–23.8)	24.2 (22.7–26.8)	<0.001
Overweight (n; %)	30/122; 24.6	11/63; 17.5	19/59; 32.2	0.059
WC (cm)	82.5 (71–92)	72 (68–79)	92 (87–96)	<0.001
WHR	0.83 (0.76–0.88)	0.76 (0.73–0.79)	0.89 (0.85–0.91)	<0.001
Abdominal obesity (n; %)	35/122; 28.7	14/63; 22.2	21/59; 35.6	0.102
SBP (mmHg)	118 (106–128)	110 (98–119)	126 (115–132)	<0.001
DBP (mmHg)	78 (68–84)	75 (66–82)	82 (73–87)	0.002
ERFE (ng/mL)	1.79 (1.19–2.61)	1.93 (1.41–3.08)	1.51 (1.06–2.3)	0.009
Fe (μmol/L)	16.4 (12.7–21.8)	14.9 (11.0–18.9)	17.9 (13.8–22.2)	0.017
Hepcidin (ng/mL)	20.1 (9.5–35.4)	12.2 (5.0–21.9)	31.7 (18.1–47.5)	<0.001
Glucose (mmol/L)	5.11 (4.83–5.33)	4.94 (4.72–5.17)	5.22 (4.94–5.44)	<0.001
CRP (mg/L)	0.60 (0.40–1.50)	0.50 (0.30–1.80)	0.75 (0.40–1.40)	0.374
HbA1c (mmol/mol)	33 (31–34)	32 (31–34)	33 (31–37)	0.092
Insulin (µU/mL)	7.29 (5.32–10.01)	6.40 (4.68–8.14)	7.94 (6.11–11.21)	0.002
HOMA-IR	1.66 (1.18–2.18)	1.41 (1.11–1.79)	1.89 (1.34–2.54)	<0.001
TC (mmol/L)	4.89 (4.40–5.38)	191 (4.40–5.30)	4.89 (4.32–5.40)	0.822
HDL-C (mmol/L)	1.40 (1.16–1.58)	1.55 (1.34–1.73)	1.21 (1.11–1.42)	<0.001
TG (mmol/L)	0.92 (0.70–1.35)	0.79 (0.61–1.08)	1.03 (0.83–1.81)	<0.001
LDL-C (mmol/L)	2.97 (2.48–3.49)	2.87 (2.38–3.21)	3.10 (2.61–3.67)	0.062
Non-HDL-C (mmol/L)	3.44 (2.97–4.01)	3.36 (2.82–3.78)	3.75 (3.18–4.16)	0.010
ApoAI (g/L)	1.43 (1.29–1.63)	1.56 (1.41–1.79)	1.31 (1.20–1.48)	<0.001
ApoB (g/L)	0.77 (0.66–0.88)	0.73 (0.62–0.86)	0.82 (0.72–0.91)	0.012

Results are presented as median and Q1–Q3 ranges (25th–75th percentile). Abbreviations: BMI, body mass index; WC, waist circumference; WHR, waist–hip ratio; SBP, systolic blood pressure; DBP, diastolic blood pressure; ERFE, erythroferrone; Fe, iron; CRP, C-reactive protein; HbA1c, glycated hemoglobin; HOMA-IR, Homeostatic Model Assessment for Insulin Resistance; TC, total cholesterol; HDL-C, high-density lipoprotein cholesterol; TG, triglycerides; LDL-C, low-density lipoprotein cholesterol; non-HDL-C, non-high-density lipoprotein cholesterol; apoAI, apolipoprotein AI; apoB, apolipoprotein B.

**Table 2:** Correlation coefficients between ERFE and selected variables in study group (n = 122).

Variables	All (n = 122)		Women (n = 63)		Men (n = 59)		*p*
	R	*p*	R	*p*	R	*p*	Women vs. Men
Age	−0.26	0.004	−0.21	0.104	−0.31	0.018	0.564
BMI	−0.65	<0.001	−0.55	<0.001	−0.65	0.000	0.400
WC	−0.52	<0.001	−0.57	<0.001	−0.44	0.001	0.347
WHR	−0.39	<0.001	−0.48	<0.001	−0.23	0.082	0.123
SBP	−0.18	0.044	0.20	0.117	−0.33	0.011	0.452
Fe	0.12	0.182	0.06	0.673	0.34	0.009	0.116
Hepcidin	−0.35	<0.001	−0.29	0.026	−0.29	0.024	1.000
CRP	−0.31	<0.001	−0.30	0.016	−0.24	0.069	0.728
HbA1c	−0.28	0.002	−0.31	0.012	−0.19	0.155	0.492
Insulin	−0.23	0.012	−0.10	0.451	−0.25	0.053	0.406
HOMA-IR	−0.23	0.010	−0.09	0.474	−0.25	0.054	0.376
HDL-C	0.26	0.003	0.24	0.056	0.13	0.323	0.540
TG	−0.32	<0.001	−0.27	0.032	−0.33	0.011	0.723
Non-HDL-C	−0.20	0.028	−0.06	0.621	−0.32	0.013	0.147
ApoB	−0.20	0.030	−0.13	0.332	−0.28	0.035	0.400

**Table 3:** Logistic regression analysis for ERFE as a predictor of selected cardiometabolic risk factors in study group (n = 122).

Risk Factors	Unadjusted		Adjusted for Age and Sex		Adjusted for Age, Sex and BMI	
	OR (95% CI)	*p*	OR (95% CI)	*p*	OR (95% CI)	*p*
Overweight (BMI > 25 kg/m^2^)	0.051 (0.007–0.381)	0.004	0.065 (0.020–0.210)	<0.001	---	---
Abdominal obesity (WC ≥ 80/≥94 cm, F/M)	0.372 (0.215–0.643)	<0.001	0.438 (0.249–0.769)	0.004	0.965 (0.519–1.079)	0.909
Elevated blood pressure (SBP 120–139 mmHg or DBP 70–89 mmHg)	1.152 (0.801−1.658)	0.446	1.285 (0.858–1.925)	0.655	1.697 (0.986–2.082)	0.148
CRP > 1 mg/L	0.648 (0.450–0.933)	0.020	0.658 (0.449–0.965)	0.032	0.789 (0.512–1.214)	0.281
HOMA-IR ≥ 2.0	0.584 (0.383–0.891)	0.013	0.630 (0.403–0.984)	0.042	0.731 (0.434–1.232)	0.239
TG ≥ 1.69 mmol/L	0.521 (0.286–0.948)	0.033	0.755 (0.403–1.412)	0.379	0.750 (0.361–1.559)	0.441
HDL-C < 1.16/<1.03 mmol/L (F/M)	1.011 (0.625–1.636)	0.965	1.167 (0.695–1.985)	0.560	1.662 (0.885–2.971)	0.117

Abbreviations: F, female; M, male.

## Data Availability

The data can be made available upon reasonable request—please contact the correspondence author. The data are not publicly available due to the fact that they contain information that could compromise the privacy of research participants.

## Supplementary Materials

The following supporting information can be downloaded at: https://www.mdpi.com/article/10.3390/nu17203205/s1, Figure S1: Correlation between serum ERFE and iron and hepcidin in the whole group (A), in women (B) and in men (C).

## Abbreviations

The following abbreviations are used in this manuscript:

ERFE	Erythroferrone
HbA1c	Glycated hemoglobin
HOMA-IR	Homeostatic Model Assessment for Insulin Resistance
CRP	C-reactive protein
OR	Odds ratio
MetS	Metabolic syndrome
IL-6	Interleukin-6
EPO	Erythropoietin
CTRP15	C1q/TNF-related protein 15
BMI	Body mass index
WC	Waist circumference
WHR	Waist–hip ratio
SBP	Systolic blood pressure
DBP	Diastolic blood pressure
EDTA	Ethylenediaminetetraacetic acid
Fe	Iron
LDL-C	Low-density lipoprotein cholesterol
Non-HDL-C	Non-high-density lipoprotein cholesterol
HDL-C	High-density lipoprotein cholesterol
TC	Total cholesterol
TG	Triglycerides
ApoB	Apolipoprotein B
ApoAI	Apolipoprotein AI
GLP-1	Glucagon-like protein-1
ATP	Adenosine triphosphate
BMP6	Bone morphogenetic protein-6
