# Supplementary material for: The Association of Serum Erythroferrone, a Regulator of Erythropoiesis and Iron Homeostasis, with Cardiometabolic Risk Factors in Apparently Healthy Young Adults—A Preliminary Study
Source: Nutrients. 2025 Oct 12;17(20):3205. doi: 10.3390/nu17203205 (PMC12566724; doi:10.3390/nu17203205)

Supplementary materials

Figure S1. Correlation between serum ERFE and iron and hepcidin in the whole group (A), in women (B) and in men (C).

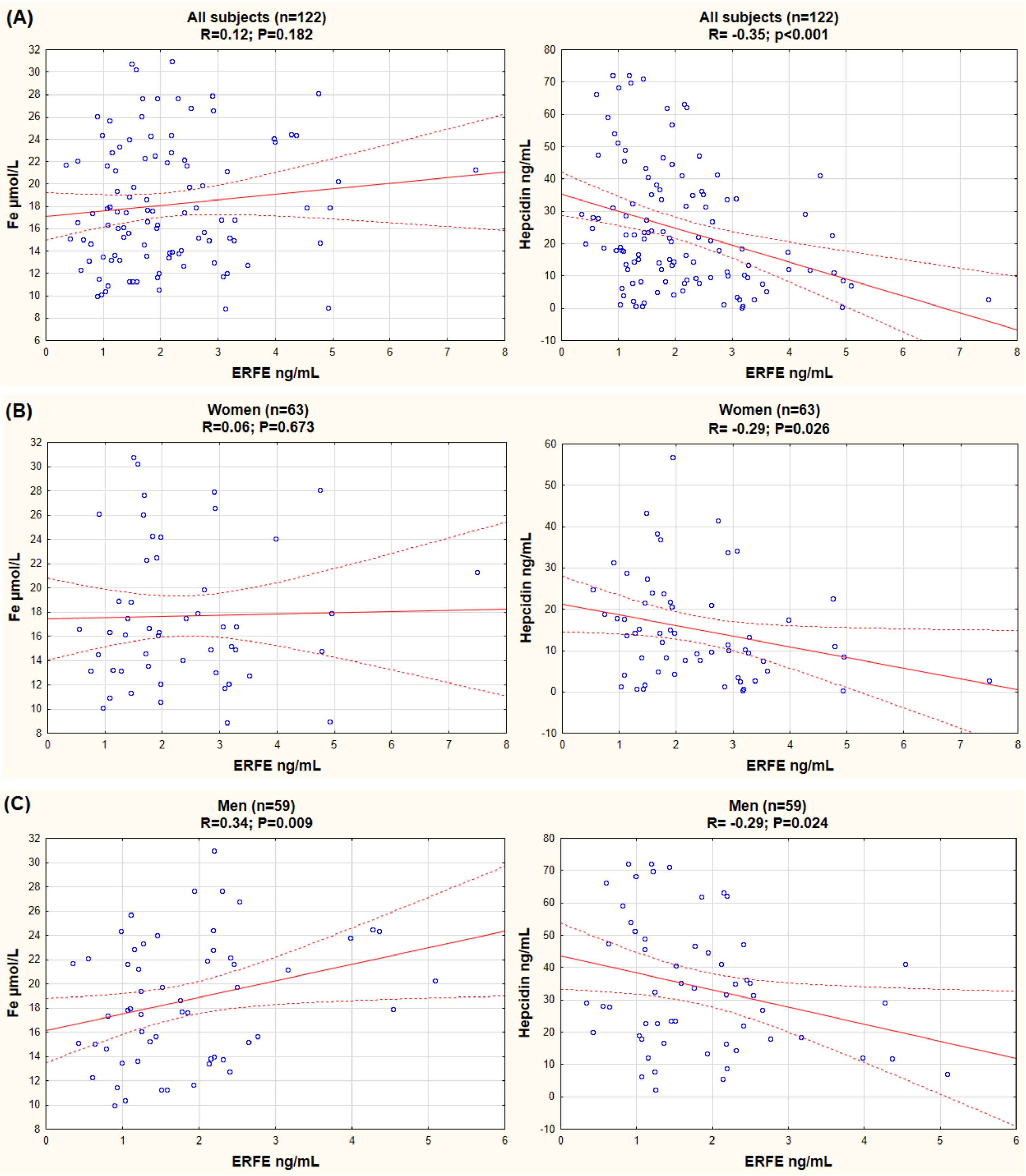

Supplement: Supplementary file 1 [file nutrients-17-03205-s001.zip › nutrients-3909943-supplementary.pdf]
